# The contribution of gut-brain axis to development of neurological symptoms in COVID-19 recovered patients: A hypothesis and review of literature

**DOI:** 10.3389/fcimb.2022.983089

**Published:** 2022-12-22

**Authors:** Kimia Vakili, Mobina Fathi, Shirin Yaghoobpoor, Fatemeh Sayehmiri, Yasaman Nazerian, Amirhossein Nazerian, Ashraf Mohamadkhani, Pariya Khodabakhsh, Gislaine Z. Réus, Ramtin Hajibeygi, Mostafa Rezaei-Tavirani

**Affiliations:** ^1^ Student Research Committee, Faculty of Medicine, Shahid Beheshti University of Medical Sciences, Tehran, Iran; ^2^ School of Medicine, Shahid Beheshti University of Medical Sciences, Tehran, Iran; ^3^ School of Medicine, Iran University of Medical Sciences, Tehran, Iran; ^4^ Digestive Disease Research Center, Tehran University of Medical Sciences, Tehran, Iran; ^5^ Department of Pharmacology, School of Medicine, Shahid Beheshti University of Medical Sciences, Tehran, Iran; ^6^ Translational Psychiatry Laboratory, Graduate Program in Health Sciences, University of Southern Santa Catarina (UNESC), Criciúma, SC, Brazil; ^7^ Department of Cardiology, Faculty of Medicine, Tehran Medical Sciences, Islamic Azad University, Tehran, Iran; ^8^ Proteomics Research Center, Faculty of Paramedical Sciences, Shahid Beheshti University of Medical Sciences, Tehran, Iran

**Keywords:** SARS-CoV-2, COVID-19, gastrointestinal microbiome, gut-brain axis, neurological disorders

## Abstract

The gut microbiota undergoes significant alterations in response to viral infections, particularly the novel SARS-CoV-2. As impaired gut microbiota can trigger numerous neurological disorders, we suggest that the long-term neurological symptoms of COVID-19 may be related to intestinal microbiota disorders in these patients. Thus, we have gathered available information on how the virus can affect the microbiota of gastrointestinal systems, both in the acute and the recovery phase of the disease, and described several mechanisms through which this gut dysbiosis can lead to long-term neurological disorders, such as Guillain-Barre syndrome, chronic fatigue, psychiatric disorders such as depression and anxiety, and even neurodegenerative diseases such as Alzheimer’s and Parkinson’s disease. These mechanisms may be mediated by inflammatory cytokines, as well as certain chemicals such as gastrointestinal hormones (e.g., CCK), neurotransmitters (e.g., 5-HT), etc. (e.g., short-chain fatty acids), and the autonomic nervous system. In addition to the direct influences of the virus, repurposed medications used for COVID-19 patients can also play a role in gut dysbiosis. In conclusion, although there are many dark spots in our current knowledge of the mechanism of COVID-19-related gut-brain axis disturbance, based on available evidence, we can hypothesize that these two phenomena are more than just a coincidence and highly recommend large-scale epidemiologic studies in the future.

## Introduction

Severe acute respiratory syndrome coronavirus 2 (SARS-CoV-2) has become a worldwide pandemic and a potential threat to global health due to its high morbidity and mortality rate. Many Coronavirus disease 2019 (COVID-19) patients experience prolonged complications after the acute phase of the disease, called “long-term COVID-19 effects”. These long-term complications should be considered a major concern affecting the morbidity and mortality of the disease. One of the most common long-term effects of COVID-19 is neurological disorders (including fatigue, anxiety, olfactory and gustatory dysfunction, dementia, depression, anxiety, memory impairment, attention disorder, obsessive-compulsive disorders, stroke, and encephalitis). Due to SARS-CoV-2 associated pathologic processes, it is predicted that infected patients are at higher risk of developing neurodegenerative diseases such as Guillain-Barré syndrome (GBS), Alzheimer′s disease (AD), and Parkinson’s disease (PD) (Hashimoto et al., 2012; Rogers et al., 2020; Taquet et al., 2021; Xu et al., 2022).

Although the pathophysiology of SARS- CoV-2-associated neurological complications is not fully understood, there are probably multiple underlying mechanisms given the variety of these symptoms (Bauer et al., 2022). Immune-mediated injury, hypoxia, coagulopathy, and viral neuroinvasion are potential processes that could be involved in the pathogenesis of these central nervous system (CNS) disorders (Zubair et al., 2020; Han et al., 2021; Jafari Khaljiri et al., 2021). Several cranial nerves innervate the human respiratory system, which serves as the main location of SARS-CoV-2 replication. The olfactory (I) and trigeminal nerve(V), the facial (VII) and glossopharyngeal (IX) nerves, as well as the vagus (X) nerve innervate the nasal cavity, upper respiratory tract, and lower respiratory tract, respectively. A growing body of evidence shows that SARS-CoV-2 may reach the CNS *via* these cranial nerves. There is also another mechanism for the virus’s neuroinvasion, which is the hematogenous route. The blood-brain barrier (BBB) must be crossed in order for the virus to disseminate hematogenously (through viremia) (Koyuncu et al., 2013; Ludlow et al., 2016).

Several studies, like those that looked at the tropism of SARS- CoV-2 along the olfactory nerve, studied the cell tropism of the virus. The olfactory bulb and choroid plexus are among many brain locations that produce the receptor angiotensin converting enzyme-2 (ACE-2) (Lukiw et al., 2020; Fodoulian et al., 2020; Chen et al., 2021). Inhibitory and excitatory neurons as well as various non-neuronal cell types, including oligodendrocytes, astrocytes, and endothelial cells, express ACE2 (Chen et al., 2021; Mahalingam et al., 2021). The ACE2/Ang (1–7)/MASR axis is considered a neuroprotective axis that prevents neurological defects *via* its anti-oxidative and anti-inflammatory functions (Xu and Lazartigues, 2020). Reduced activity of ACE2 caused by the SARS-CoV-2 active infection leads to renin-angiotensin system (RAS) dysregulation (Khodabakhsh et al., 2021). RAS dysfunction can also lead to inflammation, oxidative stress, microglial activation, and degeneration of dopaminergic neurons (Follmer, 2020; Lopez-Leon et al., 2021). Imbalance of the RAS system, over-activation of ACE/Angiotensin II (AngII)/Angiotensin II receptor type 1(AT1) pathway and dysfunctional ACE2/Ang (1-7)/Mas pathway have been related to the pathogenesis of several neurodegenerative diseases such as AD and PD, as well as multiple sclerosis (MS) (Abiodun and Ola, 2020; Li et al., 2020; Méndez-García et al., 2022). In addition to all these direct effects, SARS-CoV-2 can also indirectly target the brain and cause neuroinflammation and neurodegenerative disorders. One of the major organs that may play a crucial role in regulating neuroinflammation is the gastrointestinal (GI) tract. One of the mechanisms that play a role in the communication between the enteric nervous system (ENS) within the GI tract and the CNS is the “microbiota-gut-brain” axis (Peterson, 2020; Nagata et al., 2022).

About 70% of all the microbes in the human body exist in the gut, and 70–80% of the body’s immune cells also reside in the GI tract (Sekirov et al., 2010; Yoo and Mazmanian, 2017). The microbiota-gut-brain axis is a bi-directional communication pathway influencing immune pathways to activate inflammatory signaling. Intestinal and systemic inflammation can lead to CNS inflammation, resulting in neurodegeneration (Manosso et al., 2021). Since SARS-CoV-2 has been shown to enter host cells *via* ACE2 receptors, it can infect lung epithelial cells as a primary target. On the other hand, the GI tract, kidneys, and cardiovascular system also express ACE2, as well as the nervous system that we mentioned earlier, so they all can be non-pulmonary targets of the virus (Hamming et al., 2004a; South et al., 2020; Zhang et al., 2020). On the other hand, using drugs in COVID-19, especially co-administration, can alter gut microbiome diversity and composition.

The human microbiota is mainly composed of bacteria from the phyla Bacteroidetes, Firmicutes, Actinobacteria, and to a lesser extent, Proteobacteria. The imbalance, frequently referred to as dysbiosis, is caused by significant changes in the ratio of these species or the emergence of new bacterial groups. Fundamental characteristics of dysbiosis include a decrease in microbial diversity and an increase in Proteobacteria (Weiss and Hennet, 2017). The altered gut microbiome in SARS-CoV-2 infection is associated with susceptibility to severe disease and predisposes patients to long-term complications. Studies have shown that specific gut microbiota species in COVID-19 patients may prevent pathogen invasion by regulating immune responses and gut ACE-2 receptor expression (Geva-Zatorsky et al., 2017; Zuo et al., 2020; Wang et al., 2022). These species can increase the differentiation of Treg/Th2 anti-inflammatory cells and decrease the differentiation of pro-inflammatory Th1/Th17 cells. Therefore, the release of pro-inflammatory cytokines such as IL-6, IL-11, IL-8, and IL-12, which are the most important mediators of inflammation and cytokine storm in the disease, is reduced (Zhou et al., 2021). In most patients, this change persists even after the SARS-CoV-2 virus disappears, and subsequent respiratory symptoms resolve, which may be involved in long-term complications (Zuo et al., 2020).

Increased levels of peripheral inflammatory cytokines, including IL-6, TNF-α, CRP, IL-1β, and IL-2 in gut microbial dysbiosis, are associated with psychiatric and neurodegenerative disorders. These peripheral cytokines can reach the CNS *via* the blood-brain barrier (BBB) or through the circumventricular organs and contribute to microglia activation. Microglia-mediated inflammation and oxidative stress can also be exacerbated by SARS-CoV-2-induced RAS imbalance. Furthermore, peripheral cytokines can increase BBB permeability, and this BBB dysfunction can facilitate the effect of cytokines on the onset and/or progression of psychiatric and neurodegenerative disorders (Aktas and Aslim, 2020; Manosso et al., 2021). During the course of the disease, viral replication can occur in the GI tract for a long time, but once the infection resolves, gut microbial dysbiosis can lead to long-term symptoms (de Oliveira et al., 2021; Yeoh et al., 2021; Toledo et al., 2022). Herein, we discuss the gut microbiota-related pathways in COVID-19 pathogenesis to understand the association between intestinal dysbiosis and SARS-CoV-2-induced long-term neurological complications.

## Supporting evidence

### Gut microbiome alterations in the acute phase of COVID-19

#### Bacteriome

Human lung tissue and upper respiratory tract epithelial cells highly express ACE-2 and TMPRSS2, explaining the vulnerability of the respiratory tract to SARS-CoV-2 infection (Sungnak et al., 2020; Zhao et al., 2020). Expression of ACE-2 is detected in many other tissues, including the heart, liver, kidneys, and intestines. High levels of ACE-2 are found in the brush border of the enterocytes in the small intestine, making these cells susceptible to SARS-CoV-2 invasion (Hamming et al., 2004b; To and Lo, 2004). Detection of the virus in fecal and GI histological samples indicates the digestive tract as a possible site of viral invasion and replication (Liu et al., 2020; Xiao et al., 2020).

The entry of SARS-CoV-2 into the cell results in the downregulation of ACE2 protein expression (Silhol et al., 2020). Disruption of the enteric ACE2 axis is associated with alterations in gut microbiota composition in human and animal studies. ACE2 forms a complex with the amino acid transporter B0AT1 to mediate the uptake of neutral amino acids such as tryptophan into intestinal cells. The mammalian target of rapamycin (mTOR) is an essential sensor of intracellular nutrient availability. It can control the gut microbiota composition by regulating the expression of small intestine antimicrobial peptides (AMPs). Thus, downregulation of ACE2 protein expression reduces antimicrobial peptide secretion, resulting in gut microbiota alterations (Camargo et al., 2009; Hashimoto et al., 2012; Santisteban et al., 2016; Kim et al., 2020).

In an *in vivo* study, Sencio et al. chose Syrian hamsters because of their remarkable capacity to replicate COVID-19 manifestations. The infected animals had increased numbers of opportunistic pathogenic bacteria, including Enterobacteriaceae and Desulfovibrionaceae, decreased numbers of the beneficial bacteria Ruminococcaceae and Lachnospiraceae, which produce short-chain fatty acids (SCFAs), and decreased blood levels of SCFAs. The aforementioned results were consistent with dysbiosis since SCFAs have a dual purpose: a protective role in preserving the integrity of the intestinal epithelial barrier and controlling inflammatory response [the role of SCFAs will be thoroughly discussed later]. Supplements containing SCFAs, however, did not result in better clinical results (Sencio et al., 2022).

Sokol et al. used SARS-CoV-2-infected macaques to study primates. They subsequently noticed changes in the gut flora, which peaked on the 13th day after infection. In the upper respiratory tract, viral presence was connected with the relative abundance of the Proteobacteria, Acinetobacter, and Ruminococcaceae (Firmicutes). At the same time, Peptostreptococcaceae (particularly the Intestinibacter) was associated with the viral presence in the rectum. These bacterial strains caused blood samples to include more inflammatory markers than usual (C-reactive protein and proinflammatory cytokines). Additionally, after viral infection, chemokine production and the abundance of several species of the *Streptococcus* genus were positively correlated. The metabolism of tryptophan, a precursor to serotonin, was impacted by SARS-decreased CoV-2’s SCFA levels in macaque feces, which also drastically changed the production of several metabolites and bile acids. It is yet unknown whether the drop in SCFA levels is due to increasing host cell demands or decreased synthesis by gut bacteria. Researchers came to the conclusion that lower SCFA levels may be linked to pro-inflammatory conditions that increase vulnerability to viral respiratory infections, particularly COVID-19, and that infected macaques exhibited mild pneumonia-like symptoms (Sokol et al., 2021).

In studies on COVID-19 patients, SARS-CoV-2 infection was associated with a lower abundance of gut commensals with known immunomodulatory potentials (including several genera from the *Ruminococcaceae*, *Lachnospiraceae, and bifidobacteriacea* families, and *Faecalibacterium prausnitzii*, *Eubacterium rectale*) and a greater relative abundance of opportunistic pathogens known to cause bacteremia (such as *Clostridium hathewayi*, *Actinomyces viscosus*, and *Bacteroides nordii*) (Zuo et al., 2020; Demidowich et al., 2020; Yeoh et al., 2021). A longitudinal study on 15 COVID-19 patients suggested a correlation between the baseline abundance of *Coprobacillus*, *Clostridium ramosum*, and *Clostridium hathewayi* and COVID-19 disease severity. Some microbial species (such as *F. prausnitzii* and *Bifidobacterium bifidum*) correlated inversely with the disease severity. These alterations in the gut microbiome composition persisted even after the resolution of respiratory symptoms (Zuo et al., 2020; Yeoh et al., 2021). It is also good to mention a study by Romani et al. here, which is about gut microbiota alterations in pediatric patients with SARS-CoV-2 infection. Interestingly, they have shown that children’s gut microbiota was rich in *Faecalibacterium*. Simultaneously, they had milder symptoms during the acute phase and during the recovery phase compared to the adult population (Romani et al., 2022). These data support the hypothesis that the gut-brain axis as one of the main contributors to the development of neurological disorders in COVID-19 and Long COVID patients.

#### Virome and mycobiome

A combination of bacteria, fungi, and viruses make up the gut microbiome; However, while the bacteriome has been the subject of much recent research, little is known about the viruses (virome) and fungi (mycome) (Vemuri et al., 2020). Recent research interest has been developed toward the investigation of the function and composition of the gut virome, which serves as a rich reservoir for bacteriophages and viruses. These findings have emphasized the idea that viruses and bacteriophages play a crucial role in the composition of the bacterial microbiome and are crucial for maintaining gut homeostasis (Shkoporov et al., 2019; Gregory et al., 2020). The gut’s potential role as a reservoir for the recurrence of the disease or persistent immune cell activation is another important concept that has emerged from investigations on viral infections. For example, HIV-infected immune cells have been shown to accumulate in the gut, where they may eventually be translocated into circulation and compromise the effectiveness of antiretroviral therapy (ART) patients. Recent investigations have shown that the persistent infection of epithelial cells in the intestine can prolong the memory immune responses and prevent reinfection in COVID-19 patients. Together, these findings elucidate the intestine’s function as a viral infection reservoir and provide insight on the involvement of intestinal immune and epithelial cells in regulating host responses to viruses (Neurath et al., 2021).

Similar to bacteria and viruses, it has been shown that intestinal fungi play a role in the development of the microbiota as well as the immune system (van Tilburg Bernardes et al., 2020). According to accumulating evidence, the host immune system may be significantly influenced by the gut mycobiota, which is also linked to bacterial activity (Zhang et al., 2017; Huang et al., 2021). Recent studies have shown the direct and indirect impact of the mycobiome on numerous GI diseases (Richard and Sokol, 2019). Even though the number of fungi is smaller than the number of bacteria in the microbiota, they have a significant effect on health, particularly when they act as a reservoir of pathogens, especially in immunocompromised hosts, and as a cofactor in the development of severe infectious diseases. Although it is unknown whether COVID-19 can alter the gut mycobiome or if the course of the disease can be affected by fungal infections, few studies have been conducted, which are good to be discussed here. Zuo et al. conducted a pilot study on 30 COVID-19 patients and showed that compared to healthy individuals, the configuration of gut mycobiome of hospitalized patients was more heterogeneous (i.e., had higher interindividual dissimilarities). Additionally, they observed an increased number of opportunistic fungi, including *Candida* and *Aspergillus* genus, in COVID-19 patients during the acute phase of the disease. Specifically, the overrepresentation of *C. albicans* was observed (Zuo et al., 2020; Zuo et al., 2020). In a more recent study, Reinold et al. evaluated the fungal gut microbiota of 30 COVID-19 patients (twenty-one mild-to-moderate and nine severe) and 23 controls before receiving any treatment. Patients who tested positive for SARS-CoV-2 were distinguishable from others by the presence of pronounced fungal communities. In patients with severe COVID-19, the fungal gut microbiota was characterized by a decreased richness, evenness, and diversity, as well as a rise in the relative abundance of the Ascomycota phylum. They also found that a hallmark of severe COVID-19 was the predominance of a single fungus species (abundance >75%). The predominant species of fungi were quite diverse from patient to patient, even among the same groups (Lei et al., 2021).

### Gut microbiome alterations in the recovery phase of COVID-19

Although Newsome et al. reported no difference in gut microbial community structure between recovered patients and uninfected controls (Newsome et al., 2021), other studies suggest that dysbiotic gut microbiota in COVID-19 patients appears to remain significantly altered after recovery. The altered gut microbiome in recovered patients could influence the development of symptoms such as fatigue, dyspnea, and joint pain in a subset of recovered patients (Yeoh et al., 2021; Tian et al., 2021). In an original study conducted on seven males who recovered from COVID-19 infection, sampling was carried out with an average time of 3 months after hospital discharge; the results showed differences in the proportion of some bacteria between recovered patients and controls. On the phylum level, COVID-19 groups showed an evident decrease in the *Actinobacteriota* phylum and an increase in Bacteroidota, Fusobacteriota, and Proteobacteria phyla. At the genus level, the COVID-19 group had elevated levels of *Rothia* and *Erysipelatoclostridium*, suggesting a possible shift of microbes from the mouth and respiratory tract to the gut. Despite the decrease in SCFA-producing bacteria in the COVID-19 group (such as Family *Ruminococcaceae*, genus *Faecalibacterium*, and *Eubacterium hallii* group), the SCFA levels of recovered patients were normal. This study has significant limitations. First, the sample size was too small, and the focus was primarily male patients. In general, due to the patient’s lack of interest or other technical limitations, it is hard to perform a longitudinal study on such cases, which justifies the small sample size of the conducted studies. Such studies offer circumstantial evidence that had to be handled very cautiously. Therefore, multicenter research involving additional cases is needed to confirm the findings. Second, only cured patients were assessed in this cross-sectional study. To fully understand the impact of COVID-19, it is helpful to look at the microbiota composition at earlier time points, for example, during hospitalization or even before SARS-CoV-2 infection. Third, because there are not enough clinical investigations, it is unknown whether an unbalanced gut flora directly causes functional problems in recovered individuals or is just a coincidence (Tian et al., 2021).

Ferreira-Junior et al. evaluated the intestinal microbiota of 149 Brazilian COVID-19-recovered patients in several periods after the disease (1 to 8 months). Total anti-SARS-CoV-2 antibodies, C-reactive protein, and DNA isolated from stool samples were measured. Significant variations in the microbiota diversity between post-COVID-19 patients and controls showed gut dysbiosis even months after the acute illness had resolved. They found fewer beneficial microorganisms, such as *Bifidobacterium* and *Akkermansia*, as well as specific genera, including *Desulfovibrio*, *Haemophillus*, *Dialister*, and *Prevotella*, probably due to antibiotic-induced and/or post-COVID-19 dysbiosis. They even hypothesized that post-COVID-19 clinical symptoms might be related to dysbiosis and the indiscriminate use of antibiotics during the pandemic. Anxiety, depression, memory loss, dyspnea, myalgia, alopecia, and fatigue were among the symptoms that 39% (n = 58) of the patients in their research experienced (Ferreira-Junior et al., 2022).

### Long-term neurological manifestation of COVID-19

As the number of COVID-19 patients is on the rise, survivors of this infection experience long-lasting complications and post-viral manifestations, such as severe acute respiratory syndrome (SARS) and the Middle East respiratory syndrome (MERS) pandemics (Liu et al., 2020). Post-COVID manifestations that used to be known as “long COVID” are now also called “post-acute sequelae of SARS-CoV-2 infection” (PASC) (Moghimi et al., 2021). Following acute SARS-CoV-2 infection, the neurocognitive and systemic symptoms of COVID-19 can be present for several weeks and potentially cause permanent impairments (Mansueto, 2020; Al-Aly et al., 2021). Various organs such as the heart, musculoskeletal system, lung, GI tract, kidneys, and brain may be responsible for the long-term manifestations (Vickers, 2017; Divani et al., 2020; Andalib et al., 2021).

Premraj et al. conducted a meta-analysis to examine the prevalence of neurological and neuropsychiatric symptoms in COVID-19-recovered patients three months (or more) after the acute phase of the disease. Nineteen studies, totalling 11,324 patients, entered the meta-analysis out of 1458 papers. Brain fog, memory problems, attention deficit, myalgia, anosmia, dysgeusia, and headache were the most prevalent neurological post-COVID-19 symptoms, orderly. Sleep problems, anxiety, and depression were among the most prevalent neuropsychiatric post-COVID-19 disorders. The prevalence of neuropsychiatric symptoms significantly increased between mid- and long-term follow-ups. Individuals hospitalized with acute COVID-19 exhibited lower rates of anosmia, dysgeusia, depression, anxiety, fatigue, headaches, myalgia, and sleep disruption three months after the acute phase of the disease compared to non-hospitalized patients. On the other hand, hospital stay was linked to a greater incidence of memory problems. The incidence of fatigue, anxiety, depressive symptoms, and sleep disruptions was greater in cohorts with >20% ICU admission rate during acute COVID-19 than in cohorts with <20% ICU admission rate. Their findings suggest that post-COVID-19 syndrome is characterized by fatigue, cognitive impairment (brain fog, memory problems, attention deficit), and sleep abnormalities. Sleep problems, depression, and anxiety are frequent psychiatric symptoms that become progressively more prevalent with time (Premraj et al., 2022).

Several mechanisms can play a role in PASC; in particular, the virus can invade the brain directly or indirectly. In addition, the immune reaction increases cytokines levels and thus can cause chronic inflammation. Other possible mechanisms are hormonal disturbances, immune dysregulation, direct organ damage, and the persistence of mild infection (Moghimi et al., 2021). There is evidence that several significant neurological symptoms and complications may persist after infection (Swain et al., 2021). It is reported by Rogers et al. that fatigue, anxiety, olfactory and gustatory dysfunction, myalgia, sleep disturbances, mental disorders, and post-traumatic stress syndrome were long-observed symptoms in individuals who survived COVID-19. The etiology of these manifestations is multifactorial. Possible etiologies are direct impacts of a viral infection, such as encephalitis, hypoxia and other physiological dysfunctions, cerebrovascular disorder, immunological activation, and some psychological effects of the disease, including social isolation (Alpert et al., 2020; Dicpinigaitis and Canning, 2020; Rogers et al., 2020; Havervall et al., 2021; Morin et al., 2021). In addition, some less common post-COVID neurological complications are reported, including GBS, acute transverse myelitis, irreversible hearing loss, opsoclonus-myoclonus syndrome, and cerebrovascular disorders, such as cerebral hemorrhage and vasculitis, and ischemic stroke (Scoppettuolo et al., 2020; Kilbertus, 2021; Maltezou et al., 2021; Emamikhah et al., 2021).

The most common long-standing COVID-19 symptom is fatigue, which occurs in 17.5% to 72% of hospitalized COVID-19 patients (Dixon et al., 2018; Carvalho-Schneider et al., 2021; Huang et al., 2021). This symptom can become persistent for more than seven months and lead to significant disability. Fatigue syndrome after viral infection and Myalgic Encephalomyelitis/Chronic Fatigue Syndrome (ME/CFS) have several symptoms in common. Furthermore, several immune responses against SARS-CoV-2 are similar to the ME/CFS pattern. For instance, an increase in cytokines, including IL-10, IL-6, and IFNγ-induced protein 10 (IP-10), is observed in their pathology. The body responds against COVID-19 the same as the ME/CFS, leading to the elevation of IP-10 levels (Dixon et al., 2018). It is shown that elevated levels of cytokines, including IL-2 and IP-10, may play a role in the presentation of long-term fatigue (Boolani and Manierre, 2019). Increased level of IP-10 is a more significant challenge to the immune system. Therefore, it can lead to more severe manifestations (Buszko et al., 2021). Leptin and resistin are increased in COVID-19 patients and cause alterations in adipocyte tissue that can exacerbate inflammatory responses in PASC (Demidowich et al., 2020; van der Voort et al., 2020; Meizlish et al., 2021). Mitochondrial impairment occurs in both ME/CFS and COVID-19, leading to a hypometabolic state and glycolysis elevation, accompanied by an increase in inflammatory reactions (Huth et al., 2020). Brain stem dysfunction, which has been shown to have roles in ME/CFS, may be responsible for PASC (Islam et al., 2020). CFS/ME has been shown to be associated with gut dysbiosis (Huang et al., 2021). COVID-19 patients who had lower microbiota richness after the convalescence have not yet fully recovered their microbiota six months after infection. Therefore, intestinal dysbiosis is related to recovery from COVID-19, and therapeutic approaches that increase microbiota diversity may hold promise in PASC treatment (Zuo et al., 2020).

There are several viruses or bacteria that can potentially stimulate GBS (Jasti et al., 2016; Berciano et al., 2020). Before the COVID-19 pandemic, there were a few cases of GBS associated with coronaviruses; however, after the COVID-19 pandemic, the number of GBS cases following SARS-CoV infection -2 has dramatically increased. The incidence of this complication of COVID-19 is higher among individuals over the age of 60 compared to individuals under the age of 40 (Trujillo Gittermann et al., 2020).

With previous viral outbreaks in mind, immune mechanisms are considered the potential factor leading to post-COVID GBS (Needham et al., 2020). Furthermore, since SARS-CoV-2 has not been detected in the CSF of most COVID-19 cases, the role of immune mechanisms is more possible than direct invasion (Iadecola et al., 2020). Due to the molecular mimicry between COVID-19 and many human tissues, it is possible for multi-organ autoimmunity in COVID-19 to cause GBS (Cao-Lormeau et al., 2016; Cappello et al., 2020; Needham et al., 2020). Furthermore, neuroinflammation is shown to play a critical role in SARS-CoV-2 effects on the nervous system. As stated, many cytokines are increased throughout this disease, including TNF- α, IL-6, IFN-γ, IL-1β, and IL-17. Many of these cytokines are responsible for the pathogenesis of GBS. Thus, COVID-19-induced cytokine storm may be a key factor in the onset and aggravation of GBS associated with COVID-19 disease (Hussain et al., 2020).

It is shown that the prognosis of most patients with the post-COVID syndrome is good, and they have no more sequelae. However, the post-COVID syndrome is not fully understood, especially regarding its long-term consequences and duration or interval (Moreno-Pérez et al., 2021).

### Microbiota-gut-brain axis in the context of COVID-19

The microbiota-gut-brain axis is a bidirectional network of signalling pathways connecting the central nervous system with the gut. Various pathways are considered for the bidirectional communication between the brain and the gut microbiota, including neural through the autonomic nervous system (ANS), neuroendocrine through the hypothalamic pituitary adrenal (HPA) axis, immune and metabolic (Khodabakhsh et al., 2021). Any alteration in the intestinal microbiota might lead to the development of behavioral changes, including depression or delirium/confusion. SARS-CoV-2 infection might affect the nervous system by altering gut microbiota composition (Premraj et al., 2022). These behavioral changes may be related to the rate of tryptophan absorption from the GI tract and the altered concentration of 5-HT in brain tissue (Ahlawat et al., 2020). The 5-HT concentration depends on many factors, such as vagus nerve activity, tryptophan levels, and metabolites of the gut, such as SCFA and bile acids (Xu et al., 2021).

A healthy intestinal mucosal barrier plays a protective role against intestinal microorganisms’ adhesion and invasion and ingresses harmful substances. Polysaccharide A (PSA) is an immunomodulatory molecule produced by *B. fragilis*. It can ameliorate symptoms of CNS demyelinating disease through the activation of regulatory T cells and the subsequent production of anti-inflammatory cytokines (Ochoa-Repáraz et al., 2010) (**Supplementary Table 1**). Unbalanced microbiota composition caused by COVID-19 can lead to increased gut permeability. Consequently, intestinal pathogens, bacterial toxins, and metabolic components can translocate to circulation and activate the immune system, resulting in increased systemic inflammation. Local and systemic exacerbated inflammation can disrupt BBB and increase its permeability. Therefore, pro-inflammatory cytokines can pass into the CNS leading to neuroinflammation (Tang et al., 2020; Manosso et al., 2021).

In many CNS disorders, such as encephalomyelitis, MS, Alzheimer’s disease (AD), and schizophrenia, the integrity of the BBB is compromised. Disruption of BBB is caused by the destruction of BBB tight junction (TJ) proteins due to the formation of reactive oxygen species (ROS) and the hyperinflammatory state. The increased permeability of BBB allows harmful substances and immune cells to enter the brain and cause CNS disorders (Daneman and Prat, 2015; Tang et al., 2020). Despite the lack of evidence for increased susceptibility to chronic neurological diseases such as PD after SARS-CoV-2 infection, it can be postulated that SARS-CoV-2 infection could predispose patients to long-term neurological disorders *via* dysfunction of ACE-2, compromising the integrity of the intestinal barrier leading to elevated levels of circulating lipopolysaccharides (LPS), and subsequent formation of α-synuclein (α-Syn) deposits in enteric nerves.

There are several pathways through which SARS-CoV-2-induced gut dysbiosis can affect the brain, and a few of them will be discussed in the following:

#### Cytokines

Increased levels of chemokines and cytokines caused by the over-activation of microglia, brain-specific macrophages, and astrocytes contribute to neuroinflammatory processes and consequent neuronal dysfunction and damage. Cytokine storm is also the major pathologic finding in COVID-19, especially the severe cases. Inflammatory cytokines can directly promote neuroinflammation *via* the autonomic nervous system (the afferent vagus nerve), which transfers signals from the intestine to the brain and indirectly *via* the cerebral endothelium (BBB). Neuro-inflammation induced by inflammatory cytokines can enhance neurodegenerative diseases *via* increasing the processing of amyloid protein, oligomerization, and deposition of Amyloid-beta (Aβ), abnormal phosphorylation and accumulation of tau, and disruption of the small vessels (Su et al., 2016; Domingues et al., 2017; Walker, 2018). Studies have shown that increased expression of IL-1, IL-6, and TNF-α is associated with Aβ plaque formation and accumulation of neurofibrillary tangles (NFTs) (Webers et al., 2020). Microglia are also involved in removing Aβ released by the neurons through degradation or releasing destructive enzymes. Reduced clearance of Aβ is shown in systemic inflammation in APP/PS1 mice (Tejera et al., 2019; Webers et al., 2020).

Increased levels of IL1β, IL2, IL6, IFNγ, and TNFα have also been detected in the striatum, serum, and ventricular or spinal cerebrospinal fluid (VCSF, LCSF) from PD patients. Higher levels of inflammatory cytokines are correlated with the extent of accumulation of α-Syn in neurons and astrocytes and consequent ROS production. Then, these inflammatory cytokines participate in α-Syn-mediated dopaminergic damage in PD patients (Nagatsu et al., 2000; Pajares et al., 2020).

MS is an autoimmune disorder and its pathology is still unknown. Multiple studies have demonstrated the role of cytokines in promoting MS. Increased transcription of genes involved in encoding inflammatory cytokines such as IL-17 and IL-6 in MS lesions and activated macrophages have been shown (Li et al., 2007). There were identified levels of IL-17R and IL-17, high levels of IL-6 in MS lesions, and high expression levels of IL-17R in MS endothelial cells involved in the transmission of neuro-inflammation mediators *via* the BBB. These inflammatory cytokines also contribute to the disruption of BBB tight junctions, which plays an essential role in the pathogenesis of multiple CNS diseases such as AD, PD, and MS (Li et al., 2007; Tzartos et al., 2008; Ireland et al., 2015; Xiao et al., 2020).

#### 5-hydroxytryptamine

Serotonin, also known as 5-hydroxytryptamine or 5-HT, acts as a significant neurotransmitter in both ends of the gut-brain axis. 5-HT is secreted by enterochromaffin cells (ECCs) and plays roles in the modulation of vagal, secretory, nociceptive, peristaltic, and vasodilatory reflexes (Crowell and Wessinger, 2007; O’Mahony et al., 2015). Within plasma, the platelets actively pick up the serotonin and store it in their dense granules, thus causing the distribution of serotonin all over the body as a hormone (O’Mahony et al., 2015). Furthermore, the serotonergic gene expression was significantly elevated as an impact of the microbiota of humanized and conventionally bred mice (Reigstad et al., 2015). The gut microbiota-derived SCFA (but not LPS) has been shown to stimulate serotonin production by human ECCs (Reigstad et al., 2015). It is indicated by Yano et al. that the gut microbiota is able to regulate the levels of 5-HT in plasma and colon tissue. The metabolites of gut microbiota, including bile acids and SCFA, can have direct effects in mediating the secretion of 5HT in ECCs (Yano et al., 2015). Szőke et al. hypothesized that serotonin, generated by intestinal ECCs, likely functions as a persistent regulatory hormonal signal for the entire body, depending on the health state of the intestine (Szőke et al., 2020). Platelets alter the permeability of different cell membranes or create free spaces between endothelial cells throughout the entire body, especially BBB (Kelly et al., 2017). According to a study by Winkler et al., serotonin can cause a short-term BBB breakdown, likely through the 5-HT2 receptors (Winkler et al., 1995). It is demonstrated that prostaglandin and unregulated ROS can mediate the effect of 5-HT on BBB disruption (Pun et al., 2009; Lochhead et al., 2010; Fraser, 2011). Therefore, this assumption has raised that ECCs-derived 5-HT, along with intestinal dysbiosis, can potentially lead to a range of human disorders, particularly neuropsychiatric and neurodevelopmental diseases (Szőke et al., 2020).

#### Cholecystokinin

CCK is a classic intestinal and cerebral peptide with various physiological effects on the GI tract and the CNS (Hadjiivanova et al., 2003). CCK is produced by ECCs of the small intestine and can affect the gut-brain axis. Gut microbiota has regulatory roles on the levels of gut peptides produced by ECCs, including CCK (Wang et al., 2020). It is demonstrated that extrinsic neural pathway activation is predominantly responsible for mediating the role of CCK in intestinal feedback. CCKR1 is a receptor on the afferent terminals of vagal nerves within the intestinal wall; this supports the paracrine and endocrine cooperation theory (Arzani et al., 2020). CCKR1 is also expressed in the hypothalamus, brain stem nuclei, and mesolimbic structures and interacts with hypothalamic hormones, serotonin, glutamate, dopamine, and neuropeptides. This justifies the CCK’s importance in extrapyramidal function, mood, and behavior (Raybould, 2007; Ballaz, 2017). Therefore, CCK can affect the gut-brain axis, and furthermore, the microbiota has regulatory effects on intestinal metabolism through this CCK-mediated gut-brain axis (Wang et al., 2020). Therefore, SARS-CoV-2, by causing alterations in the microbiota, can affect the mentioned mechanism.

#### Short-chain fatty acids

Gut commensals can reinforce intestinal barrier function and regulate the immune system through different mechanisms. The immunoregulatory effects of commensal gut bacteria are thought to occur through the secretion of metabolites, such as SCFAs, especially butyrate (Hiippala et al., 2018). Studies reported a lower relative abundance of butyrate-producing bacteria, such as several genera from the Lachnospiraceae and Ruminococcaceae families in the intestinal microbial community of COVID-19 patients; however, as mentioned previously, the reported SCFA concentrations of recovered patients were normal (Gu et al., 2020; Zuo et al., 2020; Tian et al., 2021). In addition, brain imaging studies discovered the potential interactive role of brain function and gut microbiota in different neuropsychiatric diseases. For instance, a recent investigation studied both choline and gut microbiota levels in prodromal steps of schizophrenia in the anterior cingulate cortex (He et al., 2018). Elevated relative amounts of Bacteroidales, Lactobacillales, and Clostridiales have been shown in fecal specimens from patients who were planed ultra-high-risk. Furthermore, alterations in the gut microbiota composition showed the elevated generation of SCFAs, which had been associated with elevated choline levels in the anterior cingulate cortex (He et al., 2018).

SCFAs, in addition to exerting local impacts on the colon and the peripheral tissues, are thought to have an essential role in the interaction between gut and brain microbiota. The excessive MCTs expression in the endothelial cells (Vijay and Morris, 2014) could simplify the passage of BBB by SCFAs because the uptake of SCFAs by the brain has already been shown in the rats after the administration of 14C-SCFAs to the carotid artery (Oldendorf, 1973). Although investigations on physiological SCFA levels in the brain are rare, all three metabolites can be detected in human cerebrospinal fluid (CSF) (95). Gut-Brain Communication and Short-Chain Fatty Acids demonstrate higher levels than reported in the peripheral blood (Liu et al., 2015). In addition to BBB crossing, SCFAs appear to have a vital role in retaining BBB integrity, which is closely related to the controlled routes of nutrients and molecules from the bloodstream to the brain and has a critical role in developing the brain and maintenance of CNS homeostasis. Since SCFAs can regulate BBB activity, germ-free mice demonstrate decreased expression of tight junction proteins like occludin and claudin, causing the elevated permeability of BBB from the intrauterine period to adulthood (Braniste et al., 2014). In addition, the recolonization of these mice with complex microbiota or a monocolonization with the SCFA-generating bacterial strains restores BBB integrity (Braniste et al., 2014). Likewise, a therapy of cerebrovascular endothelial cells in *in vitro* model with propionate reduces the impermeable effects of lipopolysaccharide (LPS) (Hoyles et al., ). The evidence shows that SCFAs which enter the CNS have neurological features (Silva et al., 2020). Although the exact mechanisms with a role in the activity of SCFAs on CNS are still unknown, many animal studies have demonstrated a broad impact on major behavioral and neurological procedures. They might play a role in vital stages of neurodegenerative and neurodevelopmental diseases.

#### Vagus nerve

Vagal afferent fibers originate from the different layers of the gastrointestinal wall. These fibers are not in direct contact with the microorganisms in the digestive tract and can indirectly sense intestinal metabolites and bacterial compounds through different routes, such as interaction with the EECs. In the presence of varying food components in the intestinal lumen, EECs produce and release 5-HT and gut hormones such as CCK, which activate 5-HT3 receptors and hormone receptors expressed on vagal afferent fibers, respectively, in order to modulate GI function. Bacterial products such as LPS and SCFAs can affect the vagus nerve either directly through receptors expressed on vagal afferent fibers or indirectly through TLR and receptors for bacterial products expressed on EECs. The vagus nerve activates anti-inflammatory pathways *via* its afferent and efferent fibers, resulting in decreased peripheral inflammation. The vagus nerve can inhibit pro-inflammatory M1 macrophages and increase the expression of tight junction proteins leading to decreased intestinal permeability (Bonaz et al., 2018).

#### Leaky gut syndrome and systemic inflammation

Mucin, antimicrobial peptides, and dimeric IgA are components of the mucosal barrier and are secreted by plasma cells, Paneth cells, and goblet cells, respectively. These effector molecules act as a barrier between luminal microorganisms and the epithelium to prevent microbial adhesion to the intestinal epithelium. However, intestinal permeability is frequently increased as a result of mucosal barrier failure (particularly the disruption of TJs), which is a pathological condition known as “leaky gut syndrome” (LGS). Inflammatory reactions are started by LGS in extraintestinal tissue as well as the intestine. A growing body of research has shown that some autoimmune disease patients have both LGS and dysbiosis (Camilleri, 2019). Dysbiosis, or changed microbial composition, has been linked to inflammatory responses and mucosal barrier dysfunction, which put the host animals at risk for systemic disorders such as obesity, inflammatory bowel disease, autoimmune diseases, food allergies, and celiac disease (Stewart et al., 2017).

Severe COVID-19 infection is characterized by elevated blood levels of pro-inflammatory markers, such as IL-6 and IL-10 (Han et al., 2020). These cytokines set the stage for dysbiosis, which changes intestinal permeability and results in leaky gut syndrome. This opens up several bacterial byproducts and toxins for further entrance, triggering a pro-inflammatory cascade. In a study including 204 COVID-19 patients, Pan et al. found that GI symptoms became worse as COVID-19 severity rose (Pan et al., 2020). In another research, individuals who had diarrhea during the acute phase of COVID-19 had higher fecal calprotectin levels (Effenberger et al., 2020). In a recent study by Prasad et al., multiple indicators of intestinal permeability were examined in the plasma (Prasad et al., 2022). When compared to healthy participants, COVID-19 patients had considerably greater levels of FABP2, PGN, and LPS, which may have been caused by the translocation of pro-inflammatory antigens from a leaky gut (Hussain et al., 2021).

### Gut-brain axis disturbance in other viral infections

The gut-brain axis consists of a variety of communication pathways between the brain and the gut. An important factor influencing these pathways is known to be the gut microbiota which affects the activity and neurochemistry of the brain (Mayer et al., 2015; Carabotti et al., 2015; Osadchiy et al., 2019). Secretions of these microbes, such as tryptophan metabolites, SCFAs, and peptidoglycans, can induce signaling pathways, affect brain immunity, cross the BBB ​​, and thus lead to communication mentioned (Cryan and Dinan, 2015; Osadchiy et al., 2019). Therefore, intestinal damage and alterations in the composition of the microbiota and intestinal dysbiosis have been shown to be correlated with the progression of neurological disorders (Miyake et al., 2015; Ma et al., 2019; Santos et al., 2019; Sochocka et al., 2019). In a normal intestine, the microbiota is preserved by an intact layer of the mucous membrane. During various viral infections, there are certain interaction mechanisms of viral bacteria in the gut (Openshaw, 2009).

#### Human immunodeficiency virus

Viral infections, including HIV (Brenchley et al., 2006), can cause injury in the gut submucosal lymphoid tissue and thus make the intestinal mucosa permeable to the microbiota and its products. Therefore, they are translocated from the gut lumen and cause malaise and similar symptoms. Another mechanism is the virus’s damage to the epithelium and the loosening of the barrier against the translocation of the microbiota (Openshaw, 2009).

TH17 is one of the lymphocytes in the Peyer’s patch that controls bacteria. HIV can infect intestinal lymphocytes, especially TH17, and thus makes the gut permeable to microbiota-derived products and subsequently elevate LPS in the bloodstream, which can stimulate immune responses and neurologic consequences, as stated (Brenchley et al., 2006; Brenchley et al., 2008).

#### Zika virus

Alterations of the gut microbiota of immunocompetent mice infected with the Zika virus (ZIKV) were evaluated by Corrêa et al. They reported that ZIKV infection increased levels of inflammation-associated bacteria, especially Deferribacteraceae, and pathogenic bacteria could emerge later. The effects of ZIKV on Enterobacteriaceae, Clostridiaceae, and Coriobacteriaceae have been shown to be an increase in their abundance. These bacterial families are functional in preserving the homeostasis and permeability of the gut. Furthermore, this infection led to damage to the colon epithelium of mice and stimulation of leukocyte infiltration (Corrêa et al., 2021).

#### Enteroviruses

Several neurotropic enteroviruses have been shown to hijack intestinal bacteria to conduct destructive attacks against the host. Therefore, they are transformed from commensal bacteria to pathogen-like. This interaction damages the gut-brain axis with fewer CNS impairments such as neuromotor, neuropsychiatric, and neurodevelopmental disorders (Clark, 2019).

#### Rotaviruses

The gut-brain axis is shown to be involved in the pathogenesis of the effects of rotaviruses on the CNS (Hellysaz and Hagbom, 2021). Rotavirus can release an enterotoxin, namely rotavirus nonstructural protein 4 (NSP4). This mediator can activate adjacent intestinal enterochromaffin cells for further release of serotonin (Hagbom et al., 2011). Thus the enteric nerves and vagal nerve to the brain are activated and can be involved in symptoms such as diarrhea and vomiting (Spiller, 2008). This represents the role of the gut-brain axis in symptoms of rotavirus infection.

#### Severe acute respiratory syndrome coronavirus

The SARS coronavirus is known as the respiratory virus; however, in addition to respiratory tract infection, it can also affect the intestinal epithelium. Despite its respiratory involvement, which can cause widespread alveolar damage, the gut alterations of this virus are milder. They can lead to high gut permeability to LPS and transmigration of the microbiota (Clark, 2019). ACE2 is a receptor that plays a role in regulating intestinal microbiota. It is indicated that in SARS-CoV infections, ACE2-mediated mechanisms can modulate intestinal inflammation (Shinu et al., 2020). LPS is a proinflammatory endotoxin that can be derived from gut bacteria. This endotoxin cannot enter the circulatory blood on normal occasions, but in conditions of increased intestinal permeability, this happens and can cause alterations in the brain. LPS may play a role as a promoter of PD and is also associated with social disturbances (Emanuele et al., 2010; Zhu et al., 2017). Injection of LPS into mice has shown its potential to cause neuroinflammation and increase Aβ in the hippocampus, memory dysfunctions, and cognitive impairments (Kahn et al., 2012; Zhao et al., 2019).

#### Influenza viruses

Influenza viruses are known to be respiratory, but some of the highly pathogenic strains can exhibit systemic and GI manifestations. Therefore, the gut is vulnerable to the influence of these strains, which can lead to increased gut permeability to the LPS in cases of severe infection. Furthermore, bacteria can cross the intestinal wall and translocate from the intestine, leading to cytokine secretion and systemic symptoms (Uiprasertkul et al., 2005; De Jong et al., 2006).

According to the evidence cited, gut-brain axis disorder can be involved in many viral infections (**Supplementary Table 1**).

### Gut microbiome alteration and drugs used for COVID-19

The relationship of the intestinal microbiota with the host is symbiotic, and the homeostasis of the host’s immune responses is related to them. Disorders of the intestinal microbiota cause damage to the immune system and, therefore, an inflammatory state in various organs, in particular, the brain, lungs, and intestines (Shahbazi et al., 2020). Impaired gut barrier function or structure has been shown to be associated with increased gut permeability and decreased tight junction protein density. As stated, this can account transmigration of bacteria or their products into the bloodstream and, thus, endotoxemia which can cause a cytokine storm (Otani and Coopersmith, 2019). It has been shown that the brain can be considered the organ most susceptible to the cytokine storm (Mishra and Banerjea, 2020).

#### Hydroxychloroquine

Hydroxychloroquine (HCQ) is known as an antimalarial drug; however, it is also used for inflammatory disorders, including systemic lupus erythematosus and rheumatoid arthritis. Since the COVID-19 outbreak, HCQ has been one of the main drugs used empirically for this infection (Otani and Coopersmith, 2019). In treating mice with HCQ, in Pan et al. study, the effects of this drug on the gut microbiota have been evaluated. They reported an increase in the abundance of Bacteroidetes and a decrease in the phylum Firmicutes. Therefore, the possibility exists that HCQ causes intestinal dysbiosis, particularly its high dose in the short term (Pan et al., 2021). Furthermore, it is shown that the gut microbiota of COVID-19 patients can be altered so that pathogenic microorganisms are increased, and the useful commensal microbiota is decreased (Zuo et al., 2020).

#### Antibiotics

Antibiotics cause many negative impacts on the intestinal microbiota, such as a reduction in microbiota diversity, changes in their metabolism, and maintenance of organisms resistant to antibiotics. These processes can result in antibiotic-associated diarrhea and recurrent infections of *Clostridioides difficile* (Ramirez et al., 2020). A study by Z.Ahmad et al. suggested that aminoglycosides, including gentamicin, have a high affinity for the major SARS-CoV-2 protease (Mpro) (Ahmed et al., 2021). However, gentamicin is shown to have potential effects on the gut microbiota. It is revealed that this antibiotic can cause a significant decline in microbiota diversity (Lei et al., 2021). Greenwood et al. demonstrated that in premature infants, gentamicin resulted in an elevation of Enterobacter in the gut microbiota (Greenwood et al., 2014).

Azithromycin is an antibiotic with potential antiviral, anti-inflammatory, and antibacterial effects. These properties suggested the potential effects of this antibiotic on COVID-19. However, its use is not supported for mild to moderate COVID-19 patients (Hinks et al., 2021). This antibiotic can lead to dysbiosis as well. A study by Li et al. showed a decrease in Lactobacillus and Rikenella in mice treated with Azithromycin (Li et al., 2017). Therefore, HCQ and antibiotics administered in COVID-19 patients can potentially cause dysbiosis and thus have adverse effects on the gut-brain axis, and evaluation of their clinical safety is needed (**Supplementary Table 1**).

#### Corticosteroids

Corticosteroids are administered for moderate, severe, or critical hospitalized COVID-19 cases who need supplemental oxygen. These drugs are immunosuppressive and anti-inflammatory and decrease the activation of macrophages, T cells, and monocytes and also reduce the production of proinflammatory cytokines and chemokines (Patel et al., 2021). A study by Mengchen Guo et al. on systemic lupus erythematosus (SLE) patients showed a significant change in microbiota diversity of patients who undergone glucocorticoid therapy (SLE+G) compared with patients without this treatment (SLE-G). The ratio of *Firmicutes* to *Bacteroidetes* in SLE+G patients was higher than the SLE-G ones. Also, *Lactobacillus*, *Bifidobacterium*, and *Enterobacteriales* levels in SLE+G group were increased (Guo et al., 2020). Jonathan D Schepper et al., in a study on mice (Schepper et al., 2020), observed that glucocorticoid treatment reduced the levels of *Bacterials* and *Verrucomicrobials*.

#### Antivirals

Tianming Dai et al. (2021) investigated the effects of some anticoronavirus drugs, including oseltamivir, darunavir, favipiravir, chloroquine, lopinavir, ribavirin, ritonavir, and arbidol on the metabolism and activity of glycosides and glycosidases of gut microbiota. Significant inhibition of the metabolism of glycosides was reported as a result of one or several of these drugs. Also, darunavir could strongly inhibit the activity of β-glucosidase.

## Hypothesis

**Figure 1:**
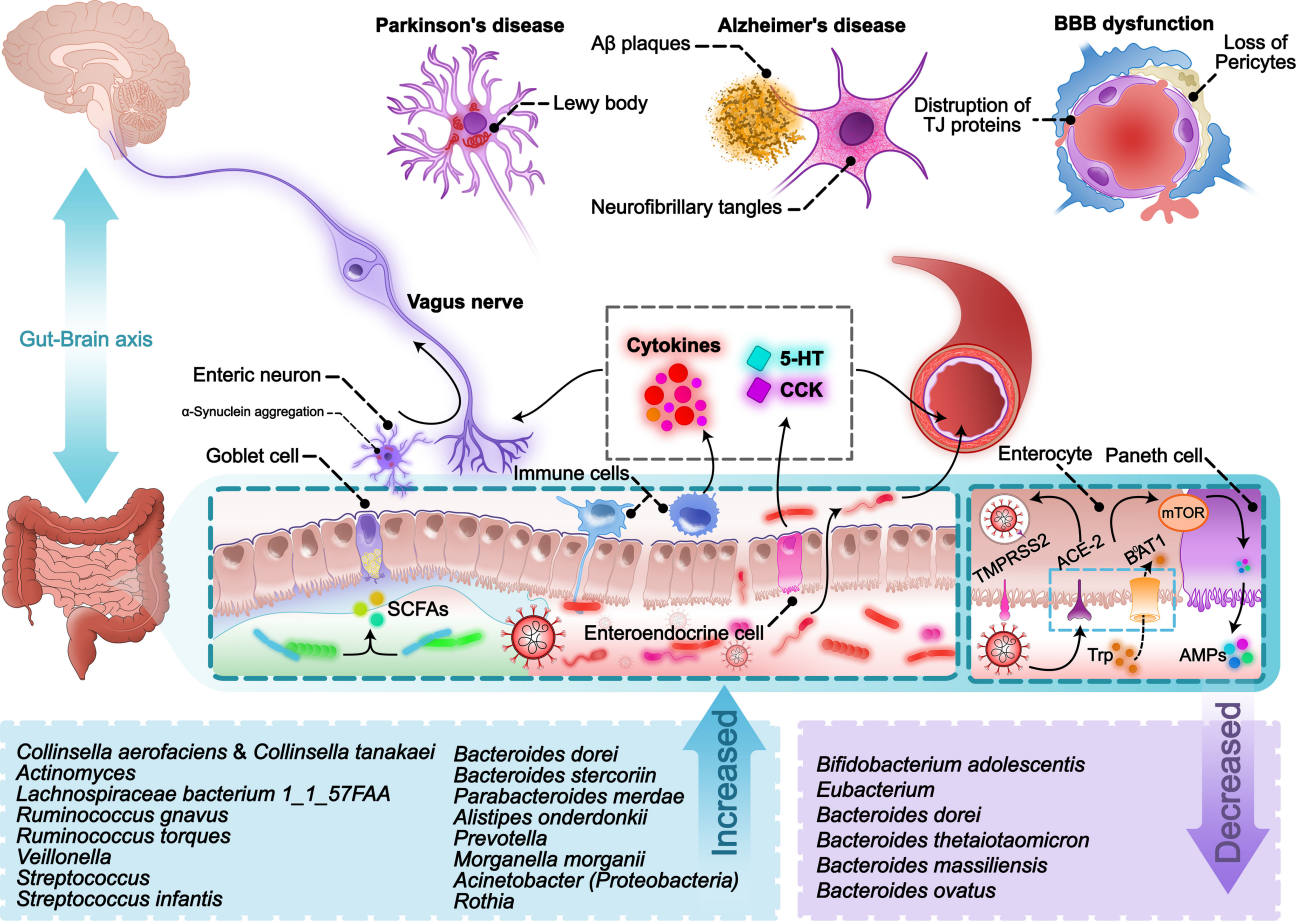
A scheme of pathways involved in SARS-CoV-2-induced gut-brain-axis disturbance.

Considering the supporting evidence, gut microbiota disturbance occurs due to acute SARS-CoV-2 infection. Unfortunately, this problem can persist even after the respiratory symptoms resolve. On the other hand, many neurological disorders are now being introduced as long-term complications of COVID-19, including chronic headache, fatigue, anxiety, olfactory and gustatory dysfunction, myalgia, sleep disturbances, neurodegenerative disorders, etc. According to the substantial body of evidence, many of these problems have previously been reported as behavioral and neurological complications caused by alteration of GI microbiota. There are also other reasons to support this claim. COVID-19 infection is not the first virus to affect gut microbiota in addition to neuro-invasive propensities. Others have been shown to be capable of altering the gut epithelium and normal microbiota. Additionally, the COVID-19 pharmacotherapeutic regimen includes numerous drugs that interfere with the normal gut microbiome. Overall, the association of SARS-CoV-2 infection and its impact on normal intestinal microorganisms with the long-term neurological complications of this disease does not seem very strange (**Figure 1**).

## Evaluation of hypothesis

To evaluate this hypothesis, we propose a prospective cohort study. In this study, all data related to the gut microbiota, history of GI disorders, history of COVID-19, history of neurological disorders and neurodegeneration, and necessary demographic information of each registered individual should be carefully collected at regular intervals.

## Discussion

Until recently, many studies have focused on acute COVID-19 infection and preventing patient death, but we still do not know what the complications of the disease will look like over time. Long-term complications of COVID-19-induced inflammation will contribute to poor outcomes and impact the quality of life. Because COVID-19 patients have a high risk of neurologic complications, detecting the possible mechanisms of neurologic defects can effectively alleviate these complications. Neurological complications caused by SARS-CoV-2 may be due to direct neuro-invasion through vagal afferents or indirect mechanisms such as neuroinflammation, possibly caused by the breakdown of the integrity of the BBB. Neuroinflammation may result from systemic inflammation caused by SARS-CoV-2-induced intestinal inflammation and gut microbiome imbalance. Hence, the microbiome-gut-brain axis is crucial for interacting with the GI tract and the CNS. There are three main pathways through which the microbiota-gut-brain axis act: 1) neuroimmune pathways, 2) neuroendocrine pathways, and 3) direct pathways of neuroinvasion, such as the vagus nerve (Mohamadkhani, 2018; Peterson, 2020; Llorens et al., 2021).

Interactions between the gut microbiome and COVID-19 have been demonstrated. Gut dysbiosis occurs during COVID-19, which is characterized by reduced microbiome diversity, decreased beneficial spices, and increased harmful species. Dysbiosis also contributes to the leaky gut syndrome and increased production of proinflammatory mediators leading to a hyperinflammatory state (Burchill et al., 2021; Hussain et al., 2021). Furthermore, the alteration of the gut microbiota persisted in the recovered patients, indicating a possible impact on the development of long-term symptoms (Tian et al., 2021). There are also other reasons for the altered composition of the gut microbiota and hyperinflammation in COVID-19. As a consequence of the binding of SARS-CoV-2 to the ACE2 receptor, downregulation of the ACE-2 receptor occurs, which leads to a reduced function of B0AT1. As mentioned, dysfunction of the B0AT1/ACE2 complex contributes to aberrant secretion of antimicrobial peptides and the alteration of gut microbiota composition.

As we know, SARS-CoV-2 enters the host cells *via* binding to the ACE2 receptor. Intestinal ACE2 functions as a chaperone for the neutral amino acid transporter B0AT1 and is essential for the superficial expression of the B0AT1 in the intestine. In the small intestine epithelium, the B0AT1/ACE2 complex contributes to the composition and function of the gut microbiota by regulating the absorption of dietary tryptophan involved in activating mTOR in mammals. Activation of mTOR influences the expression of antimicrobial peptides, and consequently, an alteration of the intestinal microbiota composition occurs. After viral entry, the downregulation of the ACE-2 receptor leads to a reduced function of B0AT1 and consequently, the aberrant secretion of antimicrobial peptides alters the composition of the gut microbiota. On the other hand, virus-induced loss of ACE2 protective functions causes an alteration of RAS signaling and Ang II accumulation after ACE-2 downregulation. This increase in Ang II levels also directly impacts gut microbial composition and metabolomics. Dysfunction of ACE2 itself is associated with the worsening of impaired gut permeability. Hence, virus-induced infection and subsequent reduced enteric ACE2 expression disrupt the blood-intestinal barrier, alters microbiota composition and metabolomics, and aggravates inflammation leading to systemic inflammation and cytokine storm (Manosso et al., 2021).

The alteration of the intestinal barrier causes the translocation of microbial metabolites such as butyrate, amyloid, and LPS. The increase in circulating LPS can lead to aberrant activity of local and systemic immunity (neuroimmune pathways). Exacerbated systemic inflammation causes an uncontrolled increase in proinflammatory cytokines (IL-6, TNF-α, CRP, IL-1β, and IL-2) known as cytokine storm, which could lead to an increase in BBB permeability, which leads to neuroinflammation, characterized by prolonged activation of microglia. There are also important signaling pathways that are involved in the integrity of BBB. The NF-κB pathway that is primarily activated in SARS-CoV-2 promotes glial cell activation and expression of ICAM-1, VCAM-1, IL-6, IL-8, and chemotactic protein 1 of monocytes (MCP-1), which all contribute to the destruction of the BBB (Tayebi and Mohamadkhani, 2012; Tang et al., 2020; Manosso et al., 2021).

On the other hand, LPS by itself can act as a promoter of neurodegenerative disorders such as AD and PD, together with its stimulatory function for the formation and aggregation of Aβ and α-Syn deposits in the enteric nerves. Enteroendocrine cells (EECs) synapse with vagus nerve terminals through the axon-like processes of EECs and are involved in alpha-synucleinopathies (direct neuroinvasion pathway). In the early stages of PD, alpha synucleinopathy is observed in the ENS prior to CNS involvement. Inflammation-related misfolding of α-Syn first occurs in the intestine, and signals which are involved in the progression of the PD transport from the gut to the CNS *via* the vagus afferent nerves. Systemic inflammation also worsens Aβ deposition by impairing the Aβ clearance capacity of the microglia *via* activation of the NLRP3 inflammasome leading to AD (Peterson, 2020; Viana et al., 2020; Manosso et al., 2021).

Another important mechanism that leads to autoimmune neurodegenerative disorders such as MS or GBS is CNS tissue-associated autoantibodies against BBB and myelin sheath. Thus, in the future, this self-attack will cause long-term neurodegenerative complications in post-COVID-19 patients (Gupta and Weaver, 2021).

Mentioned mechanisms have been considered the main mechanisms involved in the dysregulation of the bidirectional gut-brain axis in COVID-19. Increased permeability of the gut-blood barrier due to SCFA deficiency, increased microbial translocation, reduced frequency of anti-inflammatory cells, and inflammation are the principal outcomes in the gut in the dysregulated bidirectional gut-brain axis. Reduced SCFA, impaired BBB, reactivation of the microglia/astrocytes, defective neurogenesis, and neuro-inflammation are the principal outcomes in the brain in the dysregulated bidirectional gut-brain axis. The dysregulated bidirectional gut-brain axis is one of the main routes for SARS-CoV-2-associated neuroinvasion. Other routs include; 1) invasion through the peripheral nerve, 2) direct involvement through the olfactory bulb, and 3) destruction of the BBB caused by inflammation. It is proved that SARS-CoV-2-associated neurological complications may be mainly due to increased pro-inflammatory cytokines and impaired immune signal pathways in CNS (neuro-inflammation) and not the direct entry of the virus into the CNS (Johnson et al., 2021; Xu et al., 2021). All these consequences may last years beyond the initial COVID-19 illness and may lead to long-term neurological complications.

The treatment received during the acute phase of the disease is also important, as we have shown that a number of these medications can probably affect the gut microbiota and further the gut-brain axis. Of these medications, antibiotics have the most robust effect on the gut microbiota (Lange et al., 2016). Although they are not a part of standard treatment now, it should be noted as cases of misdiagnosis with bacterial infections may occur. The current evidence also shows that some of the standard treatments of the disease, such as glucocorticoids and several antivirals, can potentially affect gut dysbiosis. Still, they are not sufficient. Therefore, further observational studies on this matter are highly demanded.

In summary, viral infections can affect intestinal microbiota in various ways, particularly SARS-CoV-2 infection and its treatment-related disorders. SARS-CoV-2 neuroinvasion and long-term neurological complications of COVID-19 appeared to be associated with this altered microbiota. This association can provide valuable information on the pathophysiology of these complications, which can help prevent the progression of severe neurological complications and develop future therapeutic strategies. As COVID-19 may become endemic in the coming years, understanding the pathologic process of the disease and the long-term effects of the drugs on the gut-brain axis and CNS should be considered. However, further studies are needed to understand the subject thoroughly.

## Data availability statement

The original contributions presented in the study are included in the article/**Supplementary Material**. Further inquiries can be directed to the corresponding authors.

## Ethics statement

This study was conducted under the approval of ethics committee of Shahid Beheshti University of Medical Sciences (Ethics code: IR.SBMU.RETECH.REC.1400.786).

## Author contributions

KV conceived the presented idea. KV, MF, SY, YN and AN searched the electronic databases, collected the data, and wrote the manuscript. MR and FS supervised the findings of this work. AM, PK, GR, RH, MR and FS critically revised the manuscript. All authors contributed to the article and approved the submitted version.
