# Supplementary material for: The contribution of gut-brain axis to development of neurological symptoms in COVID-19 recovered patients: A hypothesis and review of literature
Source: Front Cell Infect Microbiol. 2022 Dec 22;12:983089. doi: 10.3389/fcimb.2022.983089 (PMC9815719; doi:10.3389/fcimb.2022.983089)
Supplement: Supplementary file 1 [file Table_1.docx]

| **Phylum** | **Class** | **Order** | **Family** | **Viral Infections** | | | **Neurologic Diseases** | | | **COVID-19 Drugs** |
| --- | --- | --- | --- | --- | --- | --- | --- | --- | --- | --- |
|  |  |  |  | **SARS-CoV-2** | **Influenza** | ***Others*** | **Alzheimer’s disease** | **Parkinson’s disease** | ***Others*** |  |
| **Actinobacteria** | **Actinobacteria** | **Actinomycetales** | **Corynebacteriaceae** | - In a study by Zuo et al. [1], a correlation between baseline abundance of *Coprobacills*, *Clostridium ramosum*, and *Clostridium hathewayi* and **COVID-19** severity was observed. |  |  | - Actinobacteria levels was increased in **AD** patients [2]. - Actinobacteria was the most abundant phylum in postmortem **AD** brain samples [3]. | - *Corynebacteriaceae* was increased in **PD** patients [4]. |  |  |
|  |  | **Bifidobacteriales** | **Bifidobacteriaceae** | - In a study by Yeoh et al. [5], a decreased *Bifidobacterium adolescentis* in gut microbiota of **COVID-19** patients was reported. | - Senico et al. reported [6] a decreased abundance of *Bifidobacteriaceae* family in fecal samples of **H3N2** infected mice. - Zhang et al. [7] have revealed a significantly increased *Bifidobacterium pseudolongum* and *Bifidobacterium animalis* within **H7N9** survived mice. |  | - *Bifidobacterium* decreased levels was observed in **AD** participants [8]. | - Bifidobacteriaceae was significantly increased in **PD** patients [9-12]. | - *Bifidobacterium* level was decreased in **GBS** patients [13]. | - Mengchen Guo et al. [14] found that in SLE patients without **Glucocorticoids** (SLE-G), Bifidobacterium level was lower in comparison to SLE patients receiving Glucocorticoid (SLE+G). - Shaodong Wei et al. [15]have observed that Bifidobacterium abundance have been decreased during the Long-term (13–39 months after the use of **azithromycin**. - Fiona Fouhy et al. have revealed that **gentamycin and ampicillin** reduce the proportion of Actinobacteria [16]. |
|  | **Coriobacteriia** | **Coriobacteriales** | **Coriobacteriaceae** | - In a study by Zuo et al. [17], an increased abundance of *Collinsella aerofaciens* and *Collinsella tanakaei* in the fecal samples **COVID-19** patients with high SARS-CoV-2 infectivity was reported. | - Sencio et al. [6] reported a decreased abundance of *Coriobacteriaceae* family in fecal samples of **H3N2** infected mice. | - Corrêa et al. [18] revealed that **Zika virus (ZIKV)** causes an increase in *Coriobacteriaceae* abundance in gut microbiota. | - Coriobacteriaceae was increased after 3, 6 and 8 months in **APP/PS1** mice [19]. | - Coriobacteriaceae was increased in **PD** patients [10, 20]. |  | - Rui Li et al. have found that **florfenicol** has decreased Gordonibacter [21]. |
|  | ***Others*** | | | - Gu et al. [22] reported a Significant increase in *Actinomyces* relative abundance in **COVID-19**. |  |  |  | - Actinomycetaciaceae. *Actinomycetales* was increased in **PD** patients [23]. |  |  |
| **Firmicutes** | **Clostridia** | **Clostridiales** | **Clostridiaceae** | - In a study by Zuo et al. [1], a correlation between *Clostridium ramosum* and *Clostridium hathewayi* abundance and **COVID-19** severity was observed. |  | - Corrêa et al. [18] revealed that **Zika virus (ZIKV)** causes an increase in *Clostridiaceae* abundance in gut microbiota. | - There is significant reductions of Firmicutes specially Clostridiaceae in APP/PS1 mouse models of **AD** and **AD** patients [8, 24-26]. | - Non-significant reduction in Clostridia was associated with severity of PD [27]. - Clostridiaceae significantly increased in PD patients [28-30]. |  | - Emmanouil Angelakis et al. have demonstrated that **doxycycline and hydroxychloroquine** can decrease the abundance of Bacteroidetes [31]. |
|  |  |  | **Lachnospiraceae** | - In a study by Yeoh et al. [5], a *Eubacterium rectale* was decreased in gut microbiota of **COVID-19** patients. - In a study by Zuo et al. [17], higher abundance of *Lachnospiraceae* bacterium 1_1_57FAA in fecal samples with none to low **SARS-CoV-2** infectivity was observed. - Gou et al. [32] reported a negative correlation between Clostridia and inflammatory markers in **COVID-19** patients. | - In a study by Groves et al. [33], a significantly decreased *Lachnospiraceae* relative abundance in mice was reported. | - In a study by Groves et al. [33], significantly decreased *Lachnospiraceae* relative abundance in **RSV**-infected mice was reported [33] | - Lanchnospiraceae was decreased in **AD** patients [2, 24] | - Lachnospiraceae was significantly reduced in **PD** patients [9, 10, 12, 20, 30]. | - The depletion of *Lachnospiraceae* occurred in **MS** and **ALS** patients [34, 35]. | - In a study by Haoqing Shao et al. [36] they have provided a diarrhea model which was induced by cefradine and gentamycin to study the microbiota features in intestinal lumen of the mice with **Antibiotic-associated diarrhea (AAD)**, the 20 top genus with high abundance were achieved. It was 5.46% for Blautia. |
|  |  |  | **Ruminococcaceae** | - Zuo et al. [1] and Yeoh et al. [5], reported an inverse correlation between *Faecalibacterium prausnitzii* abundance and **COVID-19** severity. - In a study by Yeoh et al. [5], enrichment of *Ruminococcus gnavus* and *Ruminococcus* *torques* in gut microbiota of **COVID-19** patients was observed [5]. - Gou et al. [32] identified *Ruminococcus gnavu* in **COVID-19** patients and its positive correlation with inflammatory markers [32] - Sokol et al. [37] reported an increase in relative abundance of some genera of the *Ruminococcaceae* family (*Firmicutes*) in gut microbiota of **COVID-19** patients compared to controls. | - In a study by Yitbarek et al. [38], an increased *Ruminococcus* abundance in fecal microbiota of **H9N2** infected chickens was reported. - Al Khatib et al. [39] reported a decrease in relative abundance of *Faecalibacterium prausnitzii* in **influenza (A or B)** infected patients compared to controls. |  | - The abundance of *Ruminococcaceae* was increased in **AD** patients [2, 24]. | - The abundance of *Ruminococcaceae* was significantly increased in **PD** patients [11, 28, 40]. | - The depletion of *Ruminococcaceae* occurred in **MS** patients [34]. | - In a study by Haoqing Shao et al. [36] they have provided a diarrhea model which was induced by cefradine and gentamycin to study the microbiota features in intestinal lumen of the mice with **Antibiotic-associated diarrhea (AAD)**, the 20 top genus with high abundance were achieved. It was 5.37% for Ruminococcus. - Jia Yin et al. have stated that **β-lactams (Ceftriaxone Sodium, Cefoperazone/Sulbactam and meropenem**) inhibited Papillibacter [41]. |
|  | **Negativicutes** | **Veillonellales** | **Veillonellaceae** | - Gu et al. [22] reported a significant increase in *Veillonella* relative abundance in **COVID-19**. |  |  | - The abundance of *Veillonellaceae* was significantly decreased in **AD** patients [2]. | - *Veillonellaceae was increased* in **PD** patients [42, 43]. |  |  |
|  | **Bacilli** | **Lactobacillales** | **Lactobacillaceae** |  | - In a study by Groves et al. [33], a significantly decreased *Lactobacillaceae* relative abundance in a **H1N1** infected mice was reported. - In a study by Yitbarek et al. [38], an increased *Isobaculum* abundance in fecal microbiota of **H9N2** infected chickens was observed. | - In a study by Groves et al. [33], a significantly decreased *Lactobacillaceae* relative abundance in **RSV**-infected mice was observed [33] | - The relative abundance of *Bacilli* was increased in **AD** patients [2]. | - Lactobacillaceae was significantly increased in **PD** patients [9-11, 28, 40, 44, 45]. |  | - Rui Li et al. have found that **azithromycin** has decreased Lactobacillus [21]. - In a study by Haoqing Shao et al. [36] they have provided a diarrhea model which was induced by cefradine and gentamycin to study the microbiota features in intestinal lumen of the mice with **Antibiotic-associated diarrhea (AAD)**, the 20 top genus with high abundance were achieved. It was 23.21% for Lactobacillus. - Han-Ki Park et al.[46] have demonstrated that following the administration of **azithromycin**, Lactobacillus has been disappeared in control group. - Mengchen Guo et al. [14] found that in SLE patients without **Glucocorticoids** (SLE-G), Streptococcus level was lower in comparison to SLE patients receiving Glucocorticoid (SLE+G). - Emmanouil Angelakis et al. have demonstrated that **doxycycline and hydroxychloroquine** can decrease the abundance of Lactobacillus [31]. - Fiona Fouhy et al. have revealed that **gentamycin and ampicillin** reduce the proportion of Lactobacillus [16]. |
|  |  |  | **Enterococcaceae** |  |  |  | - The relative abundance of *Enterococcaceae* was increased **AD** patients [2]. | - *Enterococcaceae* is significantly increased PD patients [10, 20, 47]. | - The enrichment of pro-inflammatory *Enterobacteriaceae* family have been reported in **MS** patients [34, 48]. | - Jia Yin et al. have shown that **Meropenem and Azithromycin** induced the growth of Enterococcus[41]. - In a study by Haoqing Shao et al. [36] they have provided a diarrhea model which was induced by cefradine and gentamycin to study the microbiota features in intestinal lumen of the mice with **Antibiotic-associated diarrhea (AAD)**, the 20 top genus with high abundance were achieved. It was for 5.97% Enterooccus. - Mengchen Guo et al. [14] found that in SLE patients without **Glucocorticoids** (SLE-G), Enterobacteriales level was lower in comparison to SLE patients receiving Glucocorticoid (SLE+G). |
|  |  | **Bacillales** | **Staphylocaccacea** |  |  |  | - *Staphylococcaceae* was more prominent in **AD** than in controls [49]. | - *staphylococci* was increased in **PD** patients [50] - Staphylococcaceae was decreased in **PD** patients [4]. |  |  |
|  | ***Others*** | | | - Zuo et al. [1] reported a correlation   between Coprobacillus abundance  and **COVID-19** severity.   - Gu et al. [22] reported a significant   increase in *Streptococcus* relative  abundance in **COVID-19**.   - In a study by Zuo et al. [17], an   increased abundance of  *Streptococcus infantis* in the fecal  Samples of **COVID-19** patients with  high SARS-CoV-2 infectivity was  reported. | - Gu et al. [22] reported an increased abundance *of Finegoldia and*   *Peptoniphilus* in **H1N1** and **H7N9**  Patients. |  | - *Gemellaceae* family and *Blautia*, *Phascolarctobacterium*, and *Gemella* genera were more abundant in **AD** participants [8]. |  |  | - In a study by Haoqing Shao et al.[36] they have provided a diarrhea model which was induced by cefradine and gentamycin to study the microbiota features in intestinal lumen of the mice with **Antibiotic-associated diarrhea (AAD)**, the 20 top genus with high abundance were achieved. It was 5.24% for Bacillus. - In a study by Haoqing Shao et al.[36] they have provided a diarrhea model which was induced by cefradine and gentamycin to study the microbiota features in intestinal lumen of the mice with **Antibiotic-associated diarrhea (AAD)**, the 20 top genus with high abundance were achieved. It was 4.62% for Eubacterium. |
| **Bacteroidetes** | **Sphingobacteriia** | **Sphingobacteriales** | **Sphingobacteriaceae** |  |  |  | - Within Bacteroidetes, *Bacteroidaceae* and *Rikenellaceae* at the family level, and *Bacteroides* and *Alistipes* at the genus level were more abundant in **AD** patients [8]. | - *Sphingobacteriacea*e was significantly reduced in **PD** patients [12]. |  | - Xiaoxian Xie et al. [51] have found that **crocin-I** at a dose of 40 mg kg−1 can maintain normal abundances of Bacteroidetes. - Yakun Sun et al.[52] found out that Bacteroidetes was increased in mice treated with **Gentamicin**. - Mengchen Guo et al. [14] found that in SLE patients without **Glucocorticoids** (SLE-G), Bacteroidetes level was increased, but the ratio of Firmicutes/Bacteroidetes has been decreased in comparison to SLE patients receiving Glucocorticoid (SLE+G). - Zhi-Yuan Pan et al. [53] have shown that **Hydroxychloroquine (HCQ)** has raised the abundance of phylum Bacteroidetes. |
|  | **Bacteroidia** | **Bacteroidales** | **Bacteroidacea** | - Zuo et al. [1] reported an inverse correlation between *Bacteroides dorei, Bacteroides thetaiotaomicron, Bacteroides massiliensis, and Bacteroides ovatus* abundance and SARS-CoV-2 load in fecal samples of patients. - In a study by Yeoh et al. [5], enrichment of *Bacteroides dorei* in gut microbiota of **COVID-19** patients was observed. - In a study by Zuo et al. [17], a higher abundance of *Bacteroides stercoriin* in fecal samples with none to low **SARS-CoV-2** infectivity was reported. | - In a study by Groves et al. [33], a significantly increased *Bacteroidaceae* relative abundance in **H1N1** infected mice was observed. - Al Khatib et al. [39] reported a higher *Bacteroides fragilis* abundance in fecal samples of **influenza A and B** shedders than non-shedders. | - In a study by Groves et al. [33], a significantly increased *Bacteroidaceae* relative abundance in RSV-infected mice was reported. | - Increased *Bacteroidaceae* was observed in **AD** patients [8]. - Zhuang, Z.-Q., et al showed that *Bacteroidia* decreased significantly in the **AD** group [2]. | - Non-significant reduction in Bacteroidia was seen in **PD** patients [27]. - Significant reduction in Bacteroidacea was seen in **PD** [44]. |  | - Emmanouil Angelakis et al. have demonstrated that **doxycycline and hydroxychloroquine** can decrease the abundance of Bacteroidetes [31] |
|  |  |  | **Tannerellaceae** | - In a study by Zuo et al. [17], a higher abundance of *Parabacteroides merdae* in fecal samples of patients with none to low **SARS-CoV-2** infectivity was reported [17] |  |  |  |  |  |  |
|  |  |  | **Rikenellacea** | - In a study by Zuo et al. [17], a higher abundance of and *Alistipes onderdonkii* in fecal samples of patients with none to low **SARS-CoV-2** infectivity was observed. |  |  | - Rikenellaceae was increased in **AD** patients [8]. | - *Rikenellaceae* was decreased in **PD** patients [12, 40, 42]. |  | - Rui Li et al. have found that **florfenicol and azithromycin** have decreased Alistipes [21]. - Jia Yin et al. have stated that **β-lactams (Ceftriaxone Sodium, Cefoperazone/Sulbactam and meropenem)** inhibited Alistipes [41]. - Rui Li et al. have found that **florfenicol and azithromycin** have decreased Rikenella [21]. |
|  |  |  | **Prevotellaceae** | association between increase in *Prevotella* in gut microbiota and COVID-19 poor prognosis and higher infectivity | - Gu et al. [22] reported an increased abundance of *Prevotella* in **H1N1** and **H7N9** patients. - Al Khatib et al. [39] reported an increase in relative abundance of *Prevotella copri* in **influenza (A or B)** infected patients compared to controls. |  | - Prevotellaceae was more abundant in APP/PS1 mice models of **AD** [54, 55]. | - Prevotellaceae was significantly reduced in **PD** patients [11, 20, 40, 56]. |  | - Jia Yin et al. have stated that **β-lactams (Ceftriaxone Sodium, Cefoperazone/Sulbactam and meropenem)** inhibited Prevotella [41]. |
|  | ***Others*** | | |  |  |  |  |  |  |  |
| **Proteobacteria** | **Gamma proteobacteria** | **Enterobacterales** | **Enterobacteriacea** |  | - Gu et al. [22] reported a higher abundance of *Escherichia-Shigella* among **H1N1** patients compared to controls and COVID-19 patients. |  | - The abundance of *Enterobacteriacea* was increased in **AD** patients [25]. | - The higher mean abundance of Gammaproteobacteria was seen in mild and severe **PD** patients [27]. |  | - Jia Yin et al. have stated that **Vancomycin** has induced the growth of Escherichia [41]. - Edward P. K. Parker et al. [57] evaluated the gut microbiota in the 6–11 month-old infants in India , receiving a 3-day period of placebo or **azithromycin** in a randomized trial which was of oral poliovirus vaccine. A remarkable decrease in the abundance of Proteobacteria in the azithromycin was seen in comparison to placebo group. - Xiaoxian Xie et al. [51] have found that **crocin-I** at a dose of 40 mg kg−1 can maintain normal abundances of Proteobacteria. - Yakun Sun et al.[52] found out that Proteobacteria was increased in mice treated with **Gentamicin**. |
|  | **Delta proteobacteria** | **Dasulfovibrionales** | **Dasulfovibrionaceae** |  |  |  | - Desulfovibrionaceae increased significantly in APP/PS1 mice models of **AD** [19]. | - Desulfovibrionaceae was increased in **PD** patients [12, 20]. | - Significant enrichment in relative abundance of members of *Desulfovibrionaceae* was observed in **MS** patients [34]. | - Mengchen Guo et al. [14] found that in SLE patients without **Glucocorticoids** (SLE-G), Bilophila level was increased in comparison to SLE patients receiving Glucocorticoid (SLE+G). - Rui Li et al. have found that **florfenicol and azithromycin** have decreased Desulfovibrio [21]. |
|  | **Epsilon proteobacteria** | **Campylobacterales** | **Helicobacteriaceae** |  |  |  | - Helicobacteraceae increased significantly in APP/PS1 mice models of **AD** [19]. | - *Helicobacter pylori* is a triggering factor in **PD** pathogenesis [58]. |  | - Armin Hinterwirth et al. have recognized that Campylobacter ureolyticus, Campylobacter jejuni, and Campylobacter hominis had been decreased in the children who were treated with **azithromycin** [59]. |
|  | ***Others*** | | | - In a study by Zuo et al. [17], an increased abundance of *Morganella morganii* in fecal samples of **COVID-19** patients with high SARS-CoV-2 infectivity was reported. - Sokol et al. [37] reported an increase in relative abundance of *Acinetobacter (Proteobacteria*) in fecal samples of **COVID-19** patients. | - In a study by Yitbarek et al. [38], an increased *Proteobacteria* abundance in fecal microbiota of **H9N2** infected chickens was observed. |  | - Proteobacteria was positively correlated with the ratio of Aβ1-42/Aβ1-40 in **AD** [25, 60]. - The *Bilophila* genus was more abundant in **AD** participants [8]. |  | - *Campylobacter jejuni* is associated with **GBS** and also **anxiety-like behavior** [61, 62]. | - Rui Li et al. have found that **florfenicol and azithromycin** have decreased Parasutterella [21]. - Fiona Fouhy et al. [16] have revealed that **gentamycin and ampicillin** reduce the proportion of Proteobacteria. - In a study by Haoqing Shao et al. [36] they have provided a diarrhea model which was induced by **cefradine and gentamycin** to study the microbiota features in intestinal lumen of the mice with Antibiotic-associated diarrhea (AAD), the 20 top genus with high abundance were achieved. It was 2.09% for Pseudomonas. |
| **Fusobacteria** | **Fusobacteriia** | **Fusobacteriales** | **Fusobacteriaceae** |  |  |  | - *Fusobacteriaceae* was decreased in **AD** patients [55]. | - Fusobacteriaceae was increased in **PD** patients [4]. |  |  |
|  | ***Others*** | | |  |  |  |  |  |  |  |
| **Verrumicrobia** | **Verrumicrobiae** | **Verrumicrobiales** | **Akkermansiaceae** |  | - Senico et al. reported [6] an increased abundance of *Verrucomicrobia* in fecal samples of **H3N2** infected mice. |  | - The abundance of *Verrucomicrobia*is declined in APPPS1 mice model of **AD** [26]. - Others studies have shown that Verrucomicrobiais and Verrucomicrobiaceae increased significantly in APP/PS1 mice models of **AD** [54, 63]. - *Akkermansia*  abundance was decreased in APP/PS1 mice models of **AD** [64]. | - Elevated levels of Verrucomicrobiae was seen in both mild and severe **PD** patients [9, 10, 27, 28, 30, 40, 43]. - The abundance of *A. muciniphila* was 80% higher in **PD** patients [28]. | - Verrucomicrobia was increased in **MS** patients [65]. | - Edward P. K. Parker et al. [57] evaluated the gut microbiota in the 6–11 month-old infants in India, receiving a 3-day period of placebo or **azithromycin** in a randomized trial which was of oral poliovirus vaccine. A remarkable decrease in the abundance of Verrucomicrobia in the azithromycin was seen in comparison to placebo group. - Jonathan D Schepper et al. [66] observe that **GC-Tx** reduced levels of Verrucomicrobials and Bacterials. |
|  | ***Others*** | | |  |  |  |  |  |  |  |
| ***Other Microorganisms*** | | | | - In a study by Yeoh et al. [5], a significant increase in Rothia relative abundance in **COVID-19** patients was reported. | - Senico et al. reported [6] an increased abundance of Cyanobacteria in fecal samples of **H3N2** infected mice. - In a study by Yitbarek et al. [38], an iIncreased *Pseudoflavonifractor, Vampirovibrio* abundance in fecal microbiota of **H9N2** infected chickens was observed. | - Corrêa et al. [18] revealed that **Zika virus (ZIKV)** causes an increase in *Deferribacteraceae* abundance in gut microbiota. |  |  |  |  |
| **References:**  1. Zuo, T., et al., *Alterations in Gut Microbiota of Patients With COVID-19 During Time of Hospitalization.* Gastroenterology, 2020. **159**(3): p. 944-955.e8.  2. Zhuang, Z.-Q., et al., *Gut Microbiota is Altered in Patients with Alzheimer’s Disease.* Journal of Alzheimer's Disease, 2018. **63**: p. 1337-1346.  3. Emery, D.C., et al., *16S rRNA Next Generation Sequencing Analysis Shows Bacteria in Alzheimer’s Post-Mortem Brain.* Frontiers in Aging Neuroscience, 2017. **9**(195).  4. Pietrucci, D., et al., *Can Gut Microbiota Be a Good Predictor for Parkinson's Disease? A Machine Learning Approach.* Brain Sci, 2020. **10**(4).  5. Yeoh, Y.K., et al., *Gut microbiota composition reflects disease severity and dysfunctional immune responses in patients with COVID-19.* Gut, 2021. **70**(4): p. 698-706.  6. Sencio, V., et al., *Gut dysbiosis during influenza contributes to pulmonary pneumococcal superinfection through altered short-chain fatty acid production.* Cell reports, 2020. **30**(9): p. 2934-2947. e6.  7. Zhang, Q., et al., *Influenza infection elicits an expansion of gut population of endogenous Bifidobacterium animalis which protects mice against infection.* Genome Biol, 2020. **21**(1): p. 99.  8. Vogt, N.M., et al., *Gut microbiome alterations in Alzheimer's disease.* Scientific reports, 2017. **7**(1): p. 13537-13537.  9. Hill-Burns, E.M., et al., *Parkinson's disease and Parkinson's disease medications have distinct signatures of the gut microbiome.* Mov Disord, 2017. **32**(5): p. 739-749.  10. Barichella, M., et al., *Unraveling gut microbiota in Parkinson's disease and atypical parkinsonism.* Mov Disord, 2019. **34**(3): p. 396-405.  11. Petrov, V.A., et al., *Analysis of Gut Microbiota in Patients with Parkinson's Disease.* Bull Exp Biol Med, 2017. **162**(6): p. 734-737.  12. Lin, A., et al., *Gut microbiota in patients with Parkinson's disease in southern China.* Parkinsonism Relat Disord, 2018. **53**: p. 82-88.  13. Shi, P., et al., *Treatment of Guillain-Barré syndrome with Bifidobacterium infantis through regulation of T helper cells subsets.* International Immunopharmacology, 2018. **61**: p. 290-296.  14. Guo, M., et al., *Alteration in gut microbiota is associated with dysregulation of cytokines and glucocorticoid therapy in systemic lupus erythematosus.* Gut microbes, 2020. **11**(6): p. 1758-1773.  15. Wei, S., et al., *Short-and long-term impacts of azithromycin treatment on the gut microbiota in children: a double-blind, randomized, placebo-controlled trial.* EBioMedicine, 2018. **38**: p. 265-272.  16. Fouhy, F., et al., *High-throughput sequencing reveals the incomplete, short-term recovery of infant gut microbiota following parenteral antibiotic treatment with ampicillin and gentamicin.* Antimicrobial agents and chemotherapy, 2012. **56**(11): p. 5811-5820.  17. Zuo, T., et al., *Depicting SARS-CoV-2 faecal viral activity in association with gut microbiota composition in patients with COVID-19.* Gut, 2021. **70**(2): p. 276-284.  18. Corrêa, R., et al., *Gut microbiota modulation induced by Zika virus infection in immunocompetent mice.* Sci Rep, 2021. **11**(1): p. 1421.  19. Shen, L., L. Liu, and H.F. Ji, *Alzheimer's Disease Histological and Behavioral Manifestations in Transgenic Mice Correlate with Specific Gut Microbiome State.* J Alzheimers Dis, 2017. **56**(1): p. 385-390.  20. Li, W., et al., *Structural changes of gut microbiota in Parkinson's disease and its correlation with clinical features.* Sci China Life Sci, 2017. **60**(11): p. 1223-1233.  21. Li, R., et al., *Effects of oral florfenicol and azithromycin on gut microbiota and adipogenesis in mice.* PloS one, 2017. **12**(7): p. e0181690.  22. Gu, S., et al., *Alterations of the Gut Microbiota in Patients With Coronavirus Disease 2019 or H1N1 Influenza.* Clin Infect Dis, 2020. **71**(10): p. 2669-2678.  23. Heintz-Buschart, A., et al., *The nasal and gut microbiome in Parkinson's disease and idiopathic rapid eye movement sleep behavior disorder.* Mov Disord, 2018. **33**(1): p. 88-98.  24. Liu, P., et al., *Altered microbiomes distinguish Alzheimer's disease from amnestic mild cognitive impairment and health in a Chinese cohort.* Brain Behav Immun, 2019. **80**: p. 633-643.  25. He, Y., et al., *Gut Microbiota: Implications in Alzheimer's Disease.* Journal of clinical medicine, 2020. **9**(7): p. 2042.  26. Harach, T., et al., *Reduction of Abeta amyloid pathology in APPPS1 transgenic mice in the absence of gut microbiota. Sci Rep 7: 41802*. 2017.  27. Gorecki, A.M., et al., *Altered Gut Microbiome in Parkinson’s Disease and the Influence of Lipopolysaccharide in a Human α-Synuclein Over-Expressing Mouse Model.* Frontiers in Neuroscience, 2019. **13**(839).  28. Baldini, F., et al., *Parkinson’s disease-associated alterations of the gut microbiome predict disease-relevant changes in metabolic functions.* BMC Biology, 2020. **18**(1): p. 62.  29. Qian, Y., et al., *Alteration of the fecal microbiota in Chinese patients with Parkinson's disease.* Brain Behav Immun, 2018. **70**: p. 194-202.  30. Keshavarzian, A., et al., *Colonic bacterial composition in Parkinson's disease.* Mov Disord, 2015. **30**(10): p. 1351-60.  31. Angelakis, E., et al., *Abnormal weight gain and gut microbiota modifications are side effects of long-term doxycycline and hydroxychloroquine treatment.* Antimicrobial agents and chemotherapy, 2014. **58**(6): p. 3342-3347.  32. Gou, W., et al., *Gut microbiota may underlie the predisposition of healthy individuals to COVID-19.* MedRxiv, 2020.  33. Groves, H.T., et al., *Respiratory Disease following Viral Lung Infection Alters the Murine Gut Microbiota.* Front Immunol, 2018. **9**: p. 182.  34. Tremlett, H., et al., *Gut microbiota in early pediatric multiple sclerosis: a case-control study.* European journal of neurology, 2016. **23**(8): p. 1308-1321.  35. Fang, X., et al., *Evaluation of the Microbial Diversity in Amyotrophic Lateral Sclerosis Using High-Throughput Sequencing.* Frontiers in microbiology, 2016. **7**: p. 1479-1479.  36. Shao, H., et al., *Gut microbiota characteristics in mice with antibiotic-associated diarrhea.* BMC microbiology, 2020. **20**(1): p. 1-9.  37. Sokol, H., et al., *SARS-CoV-2 infection in nonhuman primates alters the composition and functional activity of the gut microbiota.* Gut Microbes, 2021. **13**(1): p. 1-19.  38. Yitbarek, A., et al., *Influenza A virus subtype H9N2 infection disrupts the composition of intestinal microbiota of chickens.* FEMS Microbiol Ecol, 2018. **94**(1).  39. Al Khatib, H.A., et al., *Profiling of Intestinal Microbiota in Patients Infected with Respiratory Influenza A and B Viruses.* Pathogens, 2021. **10**(6).  40. Scheperjans, F., et al., *Gut microbiota are related to Parkinson's disease and clinical phenotype.* Mov Disord, 2015. **30**(3): p. 350-8.  41. Yin, J., et al., *Different dynamic patterns of β-lactams, quinolones, glycopeptides and macrolides on mouse gut microbial diversity.* PloS one, 2015. **10**(5): p. e0126712.  42. Pietrucci, D., et al., *Can Gut Microbiota Be a Good Predictor for Parkinson's Disease? A Machine Learning Approach.* Brain sciences, 2020. **10**(4): p. 242.  43. Vascellari, S., et al., *Gut Microbiota and Metabolome Alterations Associated with Parkinson&#x2019;s Disease.* mSystems, 2020. **5**(5): p. e00561-20.  44. Hasegawa, S., et al., *Intestinal Dysbiosis and Lowered Serum Lipopolysaccharide-Binding Protein in Parkinson's Disease.* PLoS One, 2015. **10**(11): p. e0142164.  45. Hill-Burns, E.M., et al., *Parkinson's disease and Parkinson's disease medications have distinct signatures of the gut microbiome.* Movement disorders : official journal of the Movement Disorder Society, 2017. **32**(5): p. 739-749.  46. Han-Ki, P., et al., *Altered gut microbiota by azithromycin attenuates airway inflammation in allergic asthma.* Journal of Allergy and Clinical Immunology, 2020. **145**(5): p. 1466.  47. Hopfner, F., et al., *Gut microbiota in Parkinson disease in a northern German cohort.* Brain Res, 2017. **1667**: p. 41-45.  48. Baranzini, S., et al., *The MS Microbiome Consortium (MSMC): an academic multi-disciplinary collaborative effort to elucidate the role of the gut microbiota in MS.* Multiple Sclerosis Journal, 2014. **20**(S1): p. 339.  49. Alonso, R., et al., *Infection of Fungi and Bacteria in Brain Tissue From Elderly Persons and Patients With Alzheimer’s Disease.* Frontiers in Aging Neuroscience, 2018. **10**(159).  50. Cassani, E., et al., *Use of probiotics for the treatment of constipation in Parkinson's disease patients.* Minerva gastroenterologica e dietologica, 2011. **57**(2): p. 117-121.  51. Xie, X., et al., *Crocin-I ameliorates the disruption of lipid metabolism and dysbiosis of the gut microbiota induced by chronic corticosterone in mice.* Food & function, 2019. **10**(10): p. 6779-6791.  52. Sun, Y., et al., *Gentamicin induced microbiome adaptations associate with increased BCAA levels and enhance severity of influenza infection.* Frontiers in immunology, 2020. **11**: p. 3821.  53. Pan, Z.-Y., et al., *Short-term high-dose gavage of hydroxychloroquine changes gut microbiota but not the intestinal integrity and immunological responses in mice.* Life sciences, 2021. **264**: p. 118450.  54. Chen, Y., et al., *Gut Microbiome Alterations Precede Cerebral Amyloidosis and Microglial Pathology in a Mouse Model of Alzheimer’s Disease.* BioMed Research International, 2020. **2020**: p. 8456596.  55. Harach, T., et al., *Reduction of Abeta amyloid pathology in APPPS1 transgenic mice in the absence of gut microbiota.* Sci Rep, 2017. **7**: p. 41802.  56. Bedarf, J.R., et al., *Functional implications of microbial and viral gut metagenome changes in early stage L-DOPA-naïve Parkinson’s disease patients.* Genome Medicine, 2017. **9**(1): p. 39.  57. Parker, E.P., et al., *Changes in the intestinal microbiota following the administration of azithromycin in a randomised placebo-controlled trial among infants in south India.* Scientific reports, 2017. **7**(1): p. 1-9.  58. Çamcı, G. and S. Oğuz, *Association between Parkinson's Disease and Helicobacter Pylori.* J Clin Neurol, 2016. **12**(2): p. 147-50.  59. Hinterwirth, A., et al., *Rapid Reduction of Campylobacter Species in the Gut Microbiome of Preschool Children after Oral Azithromycin: A Randomized Controlled Trial.* The American Journal of Tropical Medicine and Hygiene, 2020. **103**(3): p. 1266.  60. Bäuerl, C., et al., *Shifts in gut microbiota composition in an APP/PSS1 transgenic mouse model of Alzheimer's disease during lifespan.* Lett Appl Microbiol, 2018. **66**(6): p. 464-471.  61. Jacobs, B.C., et al., *Campylobacter jejuni infections and anti-GM1 antibodies in Guillain-Barré syndrome.* Ann Neurol, 1996. **40**(2): p. 181-7.  62. Goehler, L.E., et al., *Campylobacter jejuni infection increases anxiety-like behavior in the holeboard: possible anatomical substrates for viscerosensory modulation of exploratory behavior.* Brain Behav Immun, 2008. **22**(3): p. 354-66.  63. Zhang, L., et al., *Altered Gut Microbiota in a Mouse Model of Alzheimer's Disease.* J Alzheimers Dis, 2017. **60**(4): p. 1241-1257.  64. Ou, Z., et al., *Protective effects of Akkermansia muciniphila on cognitive deficits and amyloid pathology in a mouse model of Alzheimer’s disease.* Nutrition & Diabetes, 2020. **10**(1): p. 12.  65. Jangi, S., et al., *Alterations of the human gut microbiome in multiple sclerosis.* Nature Communications, 2016. **7**(1): p. 12015.  66. Schepper, J.D., et al., *Involvement of the gut microbiota and barrier function in Glucocorticoid‐Induced osteoporosis.* Journal of Bone and Mineral Research, 2020. **35**(4): p. 801-820. | | | | | | | | | | |
